# Why public dismissal of nutrition science makes sense: Post-truth, public accountability and dietary credibility

**DOI:** 10.1108/BFJ-10-2017-0558

**Published:** 2018-09-03

**Authors:** Bart Penders

**Affiliations:** Department of Health, Ethics and Society, Care and Public Health Research Institute, Maastricht University, Maastricht, The Netherlands

**Keywords:** Credibility, Post-truth

## Abstract

**Purpose:**

The purpose of this paper is to critically engage with societal origins of public (dis)trust and public credibility of nutrition science and offer suggestions for addressing its public dismissal.

**Design/methodology/approach:**

This viewpoint presents a conceptual analysis of public dismissal of nutrition science, drawing together perspectives on the relationships between science and society from the history, sociology and philosophy of science.

**Findings:**

The origin of trust amongst scientists relies is actively tied to their social and moral status and science as a cultural activity is inextricably linked to institutions of power. Accordingly, trust in science relies heavily on public perceptions of those institutions, the ways in which citizens feel represented by them, and to what extent citizens consider these institutions to be held accountable. Ignoring this origin leads to expectations of science and scientists they cannot live up to and inevitable disappointment in those holding such expectations.

**Social implications:**

Managing responsible expectations asks that we first dismiss dominant portrayals of science as pure, neutral, value-free and fuelled by curiosity. Second, we should pursue a reorganisation of science, favouring social inclusiveness over scientific exceptionalism.

**Originality/value:**

Post-truth dynamics are a source of concern in the dissemination of nutrition science. Rather than dismissing it as a consequence of public ignorance, a comprehensive engagement with post-truth arguments allows a constructive repositioning of nutrition science organisation and communication. It asks that we design research programmes and studies differently, incorporate different voices. Above all else, it asks humility of researchers and tolerant approaches to other perspectives.

## Trust and credibility

“As for butter vs margarine, I trust cows more than chemists”. That short quote, attributed to Joan Gussow (Gussow, quoted in Miller, 1986) offers a critical perspective on contemporary public trust in science and expertise. Its 11 words can teach us a lot about how trust emerges. The statement is not about how saturated and unsaturated fatty acids affect biomarkers or human health. It is not about any characteristic of either butter or margarine. Rather, trust in butter and distrust of margarines is tied to the origin of these spreadable fats: the technical, social, political and economic systems that gave rise to them and the characteristics we attribute to those systems.

How does something acquire our trust? Why do we trust science? And importantly, in the current socio-political climate, do we trust science less? This paper will start by going back to the origins of modern science, to the processes that gave rise to its credibility and that were meant to safeguard it. It will shed some light on the technical, social, political and economic systems that gave rise to science. It will then fast-forward to 22 April 2017 when the Marches for Science took place – in response to what many have called a credibility crisis in science, as well as opposing post-truth and post-fact narratives in the context of the so-called “War on Science” – before moving into what can be done about it. It will be mixing up history, sociology and anthropology of science for this, because these fields allow us to look at science, facts, truth and credibility in a different light.

The history of the modern science we know starts in the early seventeenth century England – the time of the scientific revolution. It is called a revolution because from that time onward, the experiment made its entry into science (Gooding *et al.*, 1989; Shapin and Schaffer, 1985). How did the young and new experimentalists of that day create credibility for their claims? To find out, we first have to understand who they were. There really were not that many scientists in the seventeenth century. There were no big national or European funding agencies supporting science, so those pursuing science had to fund themselves. More difficult still, being a scientist was not an actual career, it was more like a hobby – all scientists were in some way, amateurs. Being employed at a university at that time did not make you a scientist. It made you a teacher. So, who, in seventeenth century England had a lot of money and not much to do all day, leaving their days and cash-purses free to fiddle around with science? Amongst a few others nobility Sir Robert Boyle, Lord Kelvin, you get the picture. They were also all white and all male. Quite importantly, coming from a very small and very elite circle, they knew each other, or knew of each other or their reputation. They were noblemen, gentlemen and with that label came attributed qualities such as modesty, honesty and above all else, moral character (Daston, 1995; Shapin, 1991; Shapin and Schaffer, 1985).

Harvard historian of science Steven Shapin points at social structures and relationships allowing truth claims and their credibility to emerge, and has called it the social history of truth (Shapin, 1994). He described how scientists in those days were personally familiar with one another and visited each other for tea and biscuits, to physically witness one another’s experiments. Actual peers, actually reviewing each other’s work. When unable to attend, a testimony from another trustworthy-by-default gentleman would do.

As the amount of people practicing science slowly rose, they no longer fitted in a single salon requiring the need for innovative structures of knowledge distribution. The written account of the experiment was born – a predecessor of the scientific paper and a literary technology or innovation (Shapin and Schaffer, 1985). That account could be trusted because of its origin, because of the impeccable moral character of the gentlemen that drafted it. Accordingly, the credibility of science finds it origin in elite structures of people who drew credibility from their social standing, from class. Bluntly put, they considered themselves better than others and considered themselves trustworthy because of it. This is of course an exaggeration, and the very brief summary of this process of social truth making listed here obscures its many nuances (Allchin, 1999; Knorr-Cetina, 1991). The core message here is the realisation that the characteristics of those who make knowledge matters: their social circles, their moral character, their virtues, their titles and the systems and networks that host and support them (Fleck, 1935/1980; Kuhn, 1962/1970). It did in the seventeenth century, and it still does now. Understanding the lives of scientists, not unlike understanding cows and chemists helps us understand the basis of credibility in science.

We scientists are no longer noblemen, most of us, at least. Science has grown exponentially and we no longer all know one another personally. Familiarity and class no longer suffice as strategies to assess and weigh moral character. Our credibility is drawn from other characteristics: affiliation with peerage has given way to affiliations with respectable institutions of science. Displays of moral character and virtues have given way to detailed methodology sections and increasing movements towards open science, in an attempt to make the nobleman’s salon encapsulate the entire globe. Upper class social circles have given way to other social circles and familiarity has given way to a complex social, political, technical and epistemic organisation of science and scientific work (Hackett *et al.*, 2017; Shapin, 1995). Science became institutionalised, apparently relying on methods, procedures and standards, but in the end, the characteristics of those who make knowledge still matter; albeit less on the level of the individual and more on the collective level of how we organise science (Shapin, 2008).

Rooted in a very particular reading of this history of science, in which scientists had capital themselves and their practices were largely disconnected from the rest of society, is the picture of ideal science held by many scientists and the public alike. It contains disinterested science, independent and free of values, ideologies and politics and knowledge for knowledge’s sake. Its practitioners are somehow more moral, angelical even, in their relentless pursuit of the truth. They are not in it for the prestige or reward. Veldkamp *et al.* (2017) have called it the “storybook image of the scientist”. That storybook image of science is not only a myth. It has developed into an expectation of who we expect scientists to be, who we hope them to be.

Last spring, over half a million scientists and sympathisers took it to the streets in a large protest. Adding those that actively backed the event online and provided other types of support and expressions of sympathy, the count rises to well over a million. That made it the biggest scientific event of all times. But what was it all about? And what does it have to do with the cows, chemists and gentlemen mentioned above? The March for Science was a protest against a few things simultaneously. The most immediate and most visible were the science policy decisions Donald Trump had either made or announced, withdrawing from the Paris Agreement, defunding a series of governmental programs and a few radical staffing choices for government agencies. These immediate concerns were supplemented with more general concerns about the status of science in society and the role envisioned for science in policy in general (Abbott *et al.*, 2017; Rosenbaum, 2017; Weinberg, 2017). These concerns signalled severe risks for science. But what were those risks exactly?

## Science and politics

Well beyond a narrative of risk – a narrative of war on science has been used for decades. Trump and his policies do not exist in a vacuum and they did not come out of nowhere. The Trump administration’s position on science cannot be isolated from the so-called “War on Science” (also the “Republican War on Science”). President Bush – the second one – was already called the “anti-science” president (Sarewitz, 2009). The war on science was explicitly mentioned by those organising many of the Marches for Science in the context of a loss of public trust and public credibility of science. Science, so many of the science marchers argued, had to be put back in its rightful place, in which science informed politics but not the other way around – in other words, the role for science would be to speak truth to power. If we believe the science marchers, science literacy among the public in general and policymakers in particular and a healthy dose of respect for the hard work scientists do, should do the trick, along with a lot of funding for science, of course (Rosenbaum, 2017).

Science scholar Clark Miller has studied the *War on Science* in detail. What can be learned from his work is that debasing, critiquing, defunding or dismissing science is often – ver*y* often – not about the content of the science at all (Miller, 2017). That goes for nutrition science, climate science, vaccination, as well as for the rest. In fact, Miller argues that the *War on Science* is a political project, combatting not science, but political opponents – the established systems and structures. Science and scientists are very much part of that establishment. Scientists, their work, their products and their institutions cannot be separated from the state, from government, from politics. Miller phrases it as follows:

They [republican politicians] attack science’s forms of truth-making, its databases, and its budgets not out of a rejection of either science or truth, but as part of a coherent strategy to weaken the power of the federal agencies that rely on them.

(Miller, 2017).

In other words, science is thrown under the bus, as collateral damage in an attempt to win elections. How could that happen? How is it possible that science is so easily dismissed, or even sacrificed, for political gain? What happened to the credibility of science, that this has become possible today? Well, science has grown so inseparable from politics that it has become for all intents and purposes, a representative of the status quo. Public dismissal of science, or public distrust of experts should be seen in the context of public discontent with authorities and elites that exert power over citizens’ lives (Leenen and Penders, 2016). The *War on Science* works for Trump because it is about public discontent and social class much more than it is about science.

The Belgian cultural historian van Reybrouck (2008) argued that populist politics are merely a symptom of an underlying problem, namely that a significant portion of citizens no longer feel represented by elected officials, and in fact, no longer are represented by them. Also, equally problematically, citizens feel unable, and often are unable to hold elected officials and the system they are part of accountable. These sentiments, distrust of the establishment, of the elite, cannot be separated from the scientific infrastructure which helps to facilitate and maintain it. Many populist political campaigns across the USA and Europe – including the Trump and Brexit campaign – have voiced explicit displeasure with the status quo, and, as a consequence, of the scientific experts that are part of it. Whether we like it or not, rejecting science is a way to rebel against the establishment.

Back to the March for Science, to whose participants this rejection was unacceptable. Overall, the solution proposed there was an increased focus on the objectivity and independence of science, as well as a campaign to educate the ignorant laity of these key characteristics of science. One of the clearest examples was the statement of support for the March of Science as released by the US Association for the Advancement of Science – well known as the publisher of science magazine. It said:

[T]o protect the rights of scientists to pursue and communicate their inquiries unimpeded, expand the placement of scientists throughout the government, build public policies upon scientific evidence, and support broad educational efforts to expand public understanding of the scientific process[1].

This statement relies on a near mythical understanding of science, one that scientists and citizens often fall for and so did the AAAS and many – but not all, of course – of the marchers for science (Penders, 2017). It is what the sociologist of science Daniel Sarewitz has called “the beautiful lie”. That lie goes as follows, quoting Sarewitz who traces its origins to Vannevar Bush (1945):

Scientific progress on a broad front results from the free play of free intellects, working on subjects of their own choice, in the manner dictated by their curiosity for exploration of the unknown.

(Sarewitz, 2016)

Or, in other words: science as free from everything except itself. This is the way scientists like to portray themselves, talk about their scientific heroes and try to defend themselves against influences they do not approve. It is a position of immense luxury: to engage in a cultural activity without any serious requirement for public accountability. And it was exactly that, a lack of representation and a lack of public accountability that fuelled Trump, Brexit and the associated rise of alternative facts. When Michael Grove said that “the people have had enough of experts”, he was drawing legitimacy from this exact problem: the problem of representation and accountability in our political infrastructure, in our democracies (Van Reybrouck, 2008). Pushing back against the public dismissal of science, and against the dynamic that fuels post-truth, will require us to devote attention to how we organise our institutions – science and democracy.

A common sign, sighted at almost every march for science stated that “Science cannot be silenced”. However, a science that aims to exert influence through the independent producing of value-free truths without outside interference is easily silenced. It requires only one thing, namely that enough people stop listening. A thought experiment of what such a science completely independent from politics or society would look like has been published as the science fiction novel *Anathem* (Stephenson, 2008). It reveals a society in which scientists engage in theorizing while locked away in monasteries, completely free of influence from the outside world, but, as a consequence, also completely devout of any influence on that outside world. It offers a vision far more scary than the alternative, in which the ties between science and society are tightened, rather than severed. Sarewitz (2016) calls this a “scary lesson” and because it means letting go of a sense of control, it might very well be. To Sarewitz science can be “saved”, can be made more reliable, more valuable and more powerful, not by wrestling it free from social influences, but by inviting them in and by carefully nurturing close and intimate relationships with them. Science has to be a part of society, not artificially attempting to hover above it.

## Science dismissal as common sense

The credibility of science, to seventeenth century noblemen, originated from a social structure that was like them: rich, inbred, elitist, aristocratic and accountable to none. The credibility of science, to twenty-first century citizens is problematic, because it originates from a social structure that is nothing like them: rich, elitist and accountable to only a few but not to them. The fact that accountability is directed mostly inward means that measures of relevance, of success, or of the worth of science, have taken on strange features: counting amounts of publications, amounts of citations, or amounts of grant euros; strange and in part perverse structures of value have been built with little to no meaning beyond the cultural boundaries of science (De Rijcke *et al.*, 2016; Hammarfelt *et al.*, 2016, 2017; Müller and de Rijcke, 2017). Accountable to (almost) none, science has, over the course of many decades, managed to corrupt itself.

Here, corrupt does not mean that scientists or research managers are stealing funds or abusing power, although even that may happen. It refers to a different form of corruption, which Harvard legal scholar Lessig calls “institutional corruption”. He describes it as follows:

Institutional corruption is manifest when there is a systemic and strategic influence which is legal, or even currently ethical, that undermines the institution’s effectiveness by diverting it from its purpose or weakening its ability to achieve its purpose, including, to the extent relevant to its purpose, weakening either the public’s trust in that institution or the institution’s inherent trustworthiness.

(Lessig, 2013)

In reference to science, such systemic and strategic influences can be external. Consider, first, for instance, the flow of research funds from for-profit actors into academia, in the form of profitable public-private partnerships (Marks, 2013, 2014), clinical trials at university hospitals (Sismondo, 2008a, b) or the privatisation of parts of the academy (Williams, 1996). Second, ideological pressures may shift the research agenda, as does the pursuit of competitive research funding (Müller and de Rijcke, 2017), competition in journal or university rankings (De Rijcke *et al.*, 2016; Rushforth and de Rijcke, 2015) or supporting national policies to build competitive knowledge economies (Hessels *et al.*, 2009). Third, in response to the previous two, some research problems may be labelled as taboos (such as the study of gun-related violence) (Kellermann and Rivara, 2013), or entire disciplines can be labelled as superfluous (as happens with the humanities every so often) (Donoghue, 2008).

Pressures can also be internal, in the ways in which science organises its own reward infrastructures and performative metrics (De Rijcke *et al.*, 2016), designs perverse incentives (Edwards and Roy, 2017) prioritises methodologies over others and conceptualises its hierarchies of evidence (Murad *et al.*, 2016). Institutional corruption is not about corrupt people. It is about good people operating in a system that drives out the good by offering only poor options, a structure that dictates specific behaviours – all within the law, but not necessarily the morally preferential options given the public mission of the university.

So, summarising and exaggerating a bit: citizens are confronted with a science that is not held publically accountable, the questions it poses and attempts to answer are those of an elite they cannot question and these questions and answers do not represent citizens and their concerns. For them to dismiss science and look beyond the claims it makes – to go post-truth – is by this account, the sensible thing to do.

What would it take to save science and its credibility? What would it take to invite social, political and moral influences and structures in and carefully nurture close and intimate relationships with them? First and foremost it requires us to drop the myth, the beautiful lie, of neutral, objective, disinterested science that can and should be fuelled by curiosity and is accountable inward only. It means that we have to learn to accept that our truths are social, our facts have politics, our theories may contain traces of ideologies and that financial structures influence all of them. Science is never pure (Shapin, 2010b). That is not a threat to science’s credibility. At least, it does not have to be. If anything, it can be a service to science’s credibility (Penders and Goven, 2013). Accepting the social, political, economic and ethical traces of science makes it possible to legitimately study these traces, to identify them, to understand them and to decide, collectively, which of them may be desirable and which of them are not – something scientists or scholar cannot do alone.

The realisation that scientists cannot continue in splendid isolation is also at the core of notions such as Responsible Research and Innovation (e.g. Owen *et al.*, 2012), social robustness (e.g. Nowotny, 2003) and upstream and midstream engagement between science and society (e.g. Fisher *et al.*, 2006; Tait, 2009). Limiting engagement to communication – as many of the science marchers proposed does not counteract the issues raised above: a science not held accountable and a science chasing questions that citizens do not identify as their own. In order to realign science and society, in order to position science so that it cannot and will not be ignored, science itself has to become inclusive and embrace the social: embrace the social elements it already contains while allowing more to enter (Mayer, 2003).

## Nutritional relevance and credibility

Embracing the social in the context of nutrition science requires increased sensitisation in practice, but also on the level of political agenda-setting (Stegmaier, 2009; Penders *et al.*, 2009). The bulk of the knowledge flowing from nutrition science does not match the societal challenges of the twenty-first century. Those challenges include demographic transitions, the increasing burden of non-communicable disease and the need for environmental sustainability in agricultural production (Fardet and Rock, 2014; Penders *et al.*, 2017). This is surprising, because the aforementioned challenges are the ones scientists, not citizens articulate as relevant. Even these scientist-articulated challenges, it would appear, are not studied enough. The mismatch between nutrition science in practice, and the research agenda left unarticulated in citizens’ minds and hearts is even greater. Those questions may not overlap with the questions, methods and approaches scientists prefer, because they are not the most.

What should I have for lunch? Why does not my sandwich taste the way I remembered it? How do I live in situations of abundance? How does one organise the life of plenty? How do societies grow old healthily and above all else, happily? How do I balance health and environment? How do I lose weight? We know very little about the most pressing public issues around nutrition, although we do know that food safety, weight-loss, technology and the many ways in which nutrition, body aesthetics and sexuality shape one another, are high on the agenda (Devcich *et al.*, 2007; Grogan, 2016; Miles *et al.*, 2004). Presently, nutrition science is mirroring clinical medicine in its pursuit of evidence-based medicine, set to underpin population guidelines, product health claims and policy narratives. Key characteristics of this approach include strict interpretations of what counts as evidence and which evidence is the best, with randomised controlled trails and their systematic review at the top of the pyramid. For the purpose of the argument presented here, an important consequence of this approach is the primary valuation of evidence that has been stripped of its context, also known as reductionism (Scrinis, 2013). Of course, reductionism is a powerful strategy to answer question in relation to isolated ingredients or isolated phenomena (Katan *et al.*, 2009). In the case of some pharmaceutical chemicals, the approach seems quite obvious. However, possibly as the result of its success, exclusive emphasis on thinking in terms of substances runs the risk of becoming a dogma, hampering nutrition science’s ability to diversify its understanding of individual and public health beyond the statistical or biochemical. When researchers do venture into issues of public health in context, target prevention as a social process or study eating as a cultural phenomenon in additional to a biological one, their research is dismissed as less hard, less scientific and as a consequence, less important. They get cited less and they get funded less and accordingly, they acquire less prestige, credit and credibility (Latour and Woolgar, 1979).

For this and other reasons, the organisation of nutrition science is still strongly influenced by a reductionist focus that orients public and commercial incentives in specific directions and obscures others (Fardet and Rock, 2014; Scrinis, 2013). Furthermore, as a result of changing national and supranational governmental research policies, significant nutrition science research funding comes from the food industry. The industry is more focussed on products and nutrients than on diets and food patterns. This focus is further strengthened by the subsequent emphasis on health claims by both industries and regulators. A reductionist focus is an attempt to exclude societal influences and it works beautifully. The more reductionist the science and the more standardised the context, the less it corresponds to society and the less citizens recognise its relevance. The more reductionist and standardised a science is, the less relevant it will be (Würbel, 2000). We all know this. Randomised clinical trialsplace emphasis on internal validity at the expense of external validity. No, to really embrace social influences, nutrition science needs to actively seek and embrace the addition of new, innovative concepts, approaches and weights of evidence to adequately study the effects of nutrition on health maintenance and disease prevention in real life, in collaboration with other relevant disciplines (O’Donnell *et al.*, 2017; Parkhurst and Abeysinghe, 2016; Penders *et al.*, 2015).

The sections above argue that post-truth, post-fact or alt-fact have less to do with the truth or the fact than with the characteristics of the system from which they emerge and the ways in which it can be held accountable. So it is that system we need to address and the accountability structures that surround it. Such processes of accountability, or account-giving, exist in various forms as a reply to the expectation that one may be asked at some time to explain one’s thoughts and actions from the past (Bovens *et al.*, 2014). In that sense, accountability has always been part of science. Public accountability, the need to offer such explanations to non-experts, is another matter. When citizens are at the end of the accountability chain, it becomes part of the mechanism of how democratic societies operate both a type of work or mechanism, and a virtue attributed to responsible practices (Bovens, 2010). These processes can be organised through the setup of large bureaucratic structures that document scientific processes in detail, accessible to those citizens, for instance through complex metrics. The open science trend is an example of this. However, this accessibility is in practice low due to the size, complexity and assumed knowledgeability. As a consequence, open science exists primarily for scientists, not for citizens. Alternatively, a dialogue-based approach towards account-giving can be pursued, in which scientists and citizens increasingly become partners up to the level of co-ownership of knowledge, strategy and policy (Damgaard and Lewis, 2014).

Such an alternative approach to public accountability in nutrition science requires an alternative organisation and governance of research (Adams, 2016) and representation or politics (Voß and Freeman, 2016). Consequently, the pursuit of a trustworthy and credible nutrition science requires reciprocity in the articulation of relevance and inclusiveness through inviting other disciplines as well as non-academics to become co-creators of the new nutrition science. These non-academics can include patients, citizens, seed breeders, farmers, but also those so often dismissed: diet authors, food bloggers, writers and more (Penders *et al.*, 2017). This allows nutrition science to display to a much larger audience how its claims came into being, who was involved in making them credible and what their current status is.

This pursuit of inclusiveness requires investment in the organisation of scientific practices to enable them to resemble real life in order to reintroduce some of the familiarity science has lost over the centuries. To overcome post-truth as a problem, nutrition science needs to move post-normal (De Marchi and Ravetz, 1999; Funtowicz and Ravetz, 1995). The pursuit of this alternative, complementary, path is an ambition broadly shared. Consider, for instance, Nutrition-in-Transition, a collective of nutrition scientists, medical doctors, philosophers and social scientists, which actively pursues such innovation. Furthermore, public sociology and sociology of science has developed the rhetoric of the “Realexperiment” – interventions through which societies themselves become the object of experiments (e.g. Krohn, 2007), a school of thought currently mostly confined to German language scientific literature. More international are discussions on citizen participation in research in the context of participatory action research in global health (Adams, 2016), or though citizen panels, conferences and summits (Voß, 2016; Voß and Amelung, 2016).

Such reciprocal and inclusive research carries consequences for research design, research practice and research translation, communication and engagement for the benefit of society. How to make this happen is an experiment in itself. Such experiments invite flexible trial design, revisiting single patient case studies, intervention mapping, participatory design interventions, and many more options that move well beyond my expertise. Legitimising this alternative strategy, or approach, for nutrition science, inclusiveness, accountability and other reciprocal competences in research design, practice and dissemination have to be a given. Above all else, however, from the scientists’ point of view, it requires humility (Jasanoff, 2003, 2007). Collaboration with non-academic, non-scientific partners as co-owners of research requires all partners to more be humble with respect to their rhetoric, humble with respect to their expectations about their ability to shape the world and above all, humble with their expectations of how credible they are.

Humility is considered a virtue. It asks that we put others and their interests ahead of ourselves. That we accept that we can be held accountable by others using criteria, norms and values we did not solely decide upon ourselves. It invites critical questions from a sceptical public. Rather that educating that public, it asks that we listen to that public and they can hold us accountable for our actions. Ultimately, a nutrition science organised, governed and evaluated with these values in mind is one that has the potential of public trust and public credibility. As a consequence, this nutrition science adds immense value to society, as well as to research itself. A requirement, for “[s]cientists without credibility are culturally impotent, and science without credibility is a meaningless enterprise” (Shapin, 2010a).

## Note

1. The full text can be found at: www.aaas.org/news/aaas-and-march-science-partner-uphold-science (accessed 5 October 2017).
